# A quantitative evidence base for population health: applying utilization-based cluster analysis to segment a patient population

**DOI:** 10.1186/s12963-016-0115-z

**Published:** 2016-11-25

**Authors:** Sabine I. Vuik, Erik Mayer, Ara Darzi

**Affiliations:** 1 Institute of Global Health Innovation, Imperial College London, 10th floor, QEQM building, St Mary’s Hospital, Praed Street, London, UK; 2 Department of Surgery, Imperial College London, 10th floor, QEQM building, St Mary’s Hospital, Praed Street, London, UK

**Keywords:** Population health, Population segmentation, Care utilization

## Abstract

**Background:**

To improve population health it is crucial to understand the different care needs within a population. Traditional population groups are often based on characteristics such as age or morbidities. However, this does not take into account specific care needs across care settings and tends to focus on high-needs patients only. This paper explores the potential of using utilization-based cluster analysis to segment a general patient population into homogenous groups.

**Methods:**

Administrative datasets covering primary and secondary care were used to construct a database of 300,000 patients, which included socio-demographic variables, morbidities, care utilization, and cost. A k-means cluster analysis grouped the patients into segments with distinct care utilization, based on six utilization variables: non-elective inpatient admissions, elective inpatient admissions, outpatient visits, GP practice visits, GP home visits, and prescriptions. These segments were analyzed post-hoc to understand their morbidity and demographic profile.

**Results:**

Eight population segments were identified, and utilization of each care setting was significantly different across all segments. Each segment also presented with different morbidity patterns and demographic characteristics, creating eight distinct care user types. Comparing these segments to traditional patient groups shows the heterogeneity of these approaches, especially for lower-needs patients.

**Conclusions:**

This analysis shows that utilization-based cluster analysis segments a patient population into distinct groups with unique care priorities, providing a quantitative evidence base to improve population health. Contrary to traditional methods, this approach also segments lower-needs populations, which can be used to inform preventive interventions. In addition, the identification of different care user types provides insight into needs across the care continuum.

**Electronic supplementary material:**

The online version of this article (doi:10.1186/s12963-016-0115-z) contains supplementary material, which is available to authorized users.

## Background

Internationally, there has been a growing focus among integrated care organizations and health systems on population health [1–3]. Population health approaches aim to improve the overall health of an entire population [4, 5]. They consider health and diseases across the care pathway, from primary prevention to acute management, to identify intervention priorities [4]. With a rising chronic disease burden, understanding the determinants of health and intervening early will only become more important for health systems trying to control cost.

To be able to improve the health of a population, it is crucial to understand the specific needs of different groups within that population and organize care around these groups [2, 6, 7]. One approach to understand the needs of a population is to group people based on characteristics such as age and long-term conditions (LTCs) [4, 7, 8]. For example, the London Health Commission segments the entire London population into groups based on their morbidities and distinguishes between children, young people, adults, and older people [7]. This approach allows care priorities to be identified for specific diseases and age groups.

Although both age and the number of LTCs provide an indication of care needs, they do not reflect actual care utilization across different care settings. Since population health often aims to reduce acute care in favor of prevention, insight into the use of primary and secondary care is essential. In addition, where existing conditions are used to group people, those with no chronic conditions are left without differentiation. Yet as the primary target for prevention, this is a key population group to understand.

An alternative that does consider different care settings and differentiates patients regardless of chronic disease status, is segmentation based on care utilization. The aim of this paper was to explore the potential value of using utilization-based cluster analysis to segment a general patient population. This was achieved by first, assessing whether utilization-based cluster analysis could distinguish different groups of care users with unique population health priorities, and, second, by comparing the cluster results with traditional population groups.

## Methods

### Data

The database for this study was constructed by linking, at a patient level, English primary care records in the Clinical Practice Research Datalink (CPRD), acute care information from the Hospital Episode Statistics (HES), and the Townsend Index of Deprivation 2001 (CPRD ISAC approval under protocol 14_211R). The study used a random sample of 300,000 patients to reflect a general local population. For each patient, six care utilization variables were calculated (see Table 1). These six utilization variables were selected as they reflect different health care providers, such as hospitals, general practitioners (GPs), and pharmacies, and different types of care, such as emergency or elective care. They were created from a long-list of 14 variables, which were combined or excluded based on their correlation and clinical relation or overlap with other variables. In addition, patient characteristics including long-term conditions, age, deprivation, cost, and risk scores were extracted or calculated. For further details on the extraction and construction of the database, please refer to Additional file 1.

**Table 1:** Cluster characteristics

	Cluster								Population mean
	1	2	3	4	5	6	7	8	
Care utilization									
Number of non-elective inpatient admissions per year, mean	0.01^a^	0.01^b^	0.34^a^	0.00^a^	0.01^b^	0.44^a^	0.00^a^	0.45^a^	0.08
Number of elective inpatient admissions per year, mean	0.01^a^	0.01^a^	0.07^a^	0.13^a^	0.02^a^	0.58^a^	0.38^a^	0.34^a^	0.13
Number of outpatient attendances per year, mean	0.16^b^	0.09^b^	1.90^b^	1.65^b^	0.29^a^	5.58^a^	3.60^a^	3.99^a^	1.43
Number of GP practice visits per year, mean	0.38^a^	2.13^a^	5.54^a^	3.40^a^	6.40^a^	13.24^a^	10.06^b^	12.14^b^	5.07
Number of GP home visits per year, mean	0.00^a^	0.01^a^	0.02^b^	0.01^a^	0.03^b^	0.06^a^	0.05^a^	1.94^a^	0.06
Number of prescriptions per year, mean	0.17^a^	1.66^a^	7.40^a^	3.04^a^	21.21^a^	55.62^a^	39.78^a^	86.96^a^	15.93
Patient characteristics									
Age at end of study period, mean	36.0^a^	34.4^a^	37.8^a^	39.1^a^	53.0^a^	61.4^a^	62.1^a^	77.1^a^	45.1
Proportion in residential care, %	0.0^a^	0.0^a^	1.0^a^	0.0^a^	1.0^a^	2.0^a^	2.0^a^	16.0^a^	1.0
Predicted risk of an emergency admission in 2012, %	2.7^b^	2.8^b^	5.3^a^	3.2^a^	4.3^a^	16.2^a^	6.7^a^	22.3^a^	5.2
Townsend Deprivation Index, %									
1 (affluent)	24.1^b^	26.7^a^	21.7^c^	25.2^b^	25.8^b^	20.9^c^	24.1^b^	21.1^c^	24.7
2	22.0^c^	22.9	21.0^c^	23.7	23.8	22.0	24.1	22.4	23.0
3	21.1	20.9	20.8	20.7	20.9	21.2	21.6	22.2	21.0
4	19.3	18.1	20.9^c^	18.7	18.3	21.0^c^	18.6	20.9	19.0
5 (deprived)	13.4^b^	11.3	15.7 ^b^	11.7	11.3	14.9^c^	11.6	13.4^c^	12.4
Disease prevalence									
Number of long-term conditions, mean	0.0^a^	0.1^a^	0.3^a^	0.1^a^	0.3^a^	1.3^a^	0.7^a^	1.7^a^	0.3
Prevalence of AMI, %	0.0^c^	0.0^c^	1.7^b^	0.0^c^	0.5^a^	13.3^b^	1.9^b^	14.0^b^	1.7
Prevalence of asthma, %	0.4^a^	3.9^a^	12.0^a^	5.6^a^	15.0^a^	23.6^a^	16.8^a^	20.6^a^	9.6
Prevalence of cancer, %	0.1^b^	0.2^b^	1.7^a^	2.8^a^	1.0^a^	14.7^b^	10.9^a^	14.0^b^	3.5
Prevalence of cerebrovascular disease, %	0.0^c^	0.1^c^	2.1^a^	0.1^c^	0.7^a^	8.9^a^	1.5^a^	15.9^a^	1.5
Prevalence of congestive heart failure, %	0.0^c^	0.0^c^	0.5^a^	0.0^c^	0.2^a^	6.8^a^	0.8^a^	11.9^a^	0.9
Prevalence of COPD, %	0.1^b^	0.1^b^	1.3^a^	0.4^a^	0.9^a^	10.4^a^	3.1^a^	15.1^a^	1.8
Prevalence of dementia, %	0.0^b^	0.0^b^	0.3^b^	0.0^a^	0.2^b^	1.7^a^	0.6^a^	10.5^a^	0.5
Prevalence of diabetes, %	0.0^a^	0.3^a^	2.5^a^	0.6^a^	8.4^a^	19.4^b^	15.4^a^	20.8^b^	5.6
Prevalence of HIV/AIDS, %	0.0	0.0	0.0	0.0	0.0	0.0	0.0	0.0	0.0
Prevalence of learning disabilities, %	0.0	0.0	0.0	0.0	0.0	0.2^b^	0.1	0.3^b^	0.0
Prevalence of liver disease, %	0.0^c^	0.0^b^	0.2^b^	0.1^b^	0.0^c^	1.0^a^	0.4^c^	0.6^b^	0.2
Prevalence of mental health conditions, %	0.0^c^	0.0^c^	0.7^a^	0.1	0.1^b^	1.9^b^	0.4^a^	1.8^b^	0.3
Prevalence of paraplegia, %	0.0^c^	0.0^b^	0.2^b^	0.0^c^	0.0^b^	1.3^a^	0.1^b^	3.1^a^	0.2
Prevalence of peptic ulcer, %	0.0^b^	0.0^b^	0.5^a^	0.1^b^	0.2^b^	2.8^b^	0.9^a^	2.7^b^	0.5
Prevalence of peripheral vascular disease, %	0.0^b^	0.0^b^	0.6^a^	0.1^a^	0.3^a^	5.4^a^	1.7^a^	7.5^a^	0.9
Prevalence of renal disease, %	0.0^a^	0.2^a^	1.3^a^	0.6^a^	4.0^a^	13.9^a^	8.5^a^	24.3^a^	3.5
Prevalence of rheumatic disease, %	0.0^a^	0.1^a^	0.6^b^	0.3^a^	0.7^b^	5.4^a^	3.8^a^	6.6^a^	1.2

Legend: ^a^: Significantly different from all 7 other clusters; ^b^: Significantly different from 6 other clusters; ^c^: Significantly different from 5 other clusters; All at 0.05/7 = 0.007 significance level (Bonferroni adjustment). All variables are significantly different across clusters at a <0.000 significance level using ANOVA, Kruskal-Wallis, or Chi Square tests

### Cluster analysis

For the cluster analysis, k-means was used with an Euclidean distance as it is efficient, fast, and can handle large datasets [9]. However, k-means requires the number of clusters (k) to be determined by the user. Hierarchical methods, on the other hand, can be analyzed for the optimal cluster number but struggle with large datasets [10]. We therefore applied hierarchical cluster analysis to 10 random samples of 3000 patients to identify the optimal number of clusters. This information was then used to perform a k-means analysis of the full dataset, to create the final clusters.

The hierarchical cluster analysis was conducted using Stata 14 software [11]. Ward’s method was used as it aims to minimize the cluster sum of squares and can therefore be considered a hierarchical analogue for k-means [12]. For each of the 10 random samples the pseudo F statistic, as defined by Calinski and Harabasz [13], and the Duda and Hart Je(2)/Je(1) index [14], were calculated for 4- to 12-cluster solutions. The pseudo F statistic assesses the cluster tightness by comparing the mean sum of squares between groups to the one within groups [14]. The Duda/Hart index uses the within-cluster sum of squared distances from the mean to compare the current cluster to a potential further split. Stata presents the Duda/Hart index with a pseudo T-squared value, and a rule of thumb for deciding the number of clusters is to look for a clustering solution with a high Je(2)/Je(1) index and a corresponding low pseudo T-squared value, with high pseudo T-squared values on either side [15].

While the pseudo F statistic showed a gradual decrease for increasing cluster numbers without any discernable peaks, low pseudo T statistics with corresponding high Je(2)/Je(1) values were found for 7- to 10-cluster solutions across the 10 samples. These four solutions were then explored with a k-means analysis for the entire population in SPSS Statistics 23 [16]. The 8-cluster solution improved on the 7-cluster model, by splitting a cluster into two distinctive groups. The 9-cluster solution, however, did not create any clinically relevant additional segments. Therefore the 8-cluster solution was selected. For graphs comparing these cluster solutions, please refer to Additional file 2.

### Statistical analysis

The segments were then reviewed and profiled based on the utilization variables, as well as other characteristics such as age and morbidities. The variables were analyzed to identify whether they were statistically different across segments using a Kruskal-Wallis test for care utilization variables and the number of LTCs (which do not meet the Normality assumption), an ANOVA test for age and risk score, and a Chi square test for proportions for the morbidity variables and the Townsend scores. Variables found to differ significantly were then explored pair-wise between segments using Mann–Whitney U tests, Student t-tests, and z-tests, respectively. The significance level of 0.05 was adjusted for the pair-wise tests using the commonly used Bonferroni method, to account for the multiplicity problem that occurs when comparing multiple clusters [17, 18].

## Results

The final dataset included 298,432 patients (50.5% female and 49.5% male), with an average age of 45 years. Deprivation in the sample was lower than the national average, with 25% of people in the most affluent national quintile and only 12% in the lowest. The k-means cluster analysis produced eight clusters based on patient care utilization patterns (see Table 1). The cluster analysis aims to maximize the distance between the clustering variables, and as a result all utilization variables were statistically different across segments. However, all non-clustering variables, which were not considered to define the segments, were also found to differ significantly. Pairwise comparisons between segments showed that for some variables, including deprivation, this difference was caused by only one or two segments.

### Care user segment profiles

**Fig. 1:**
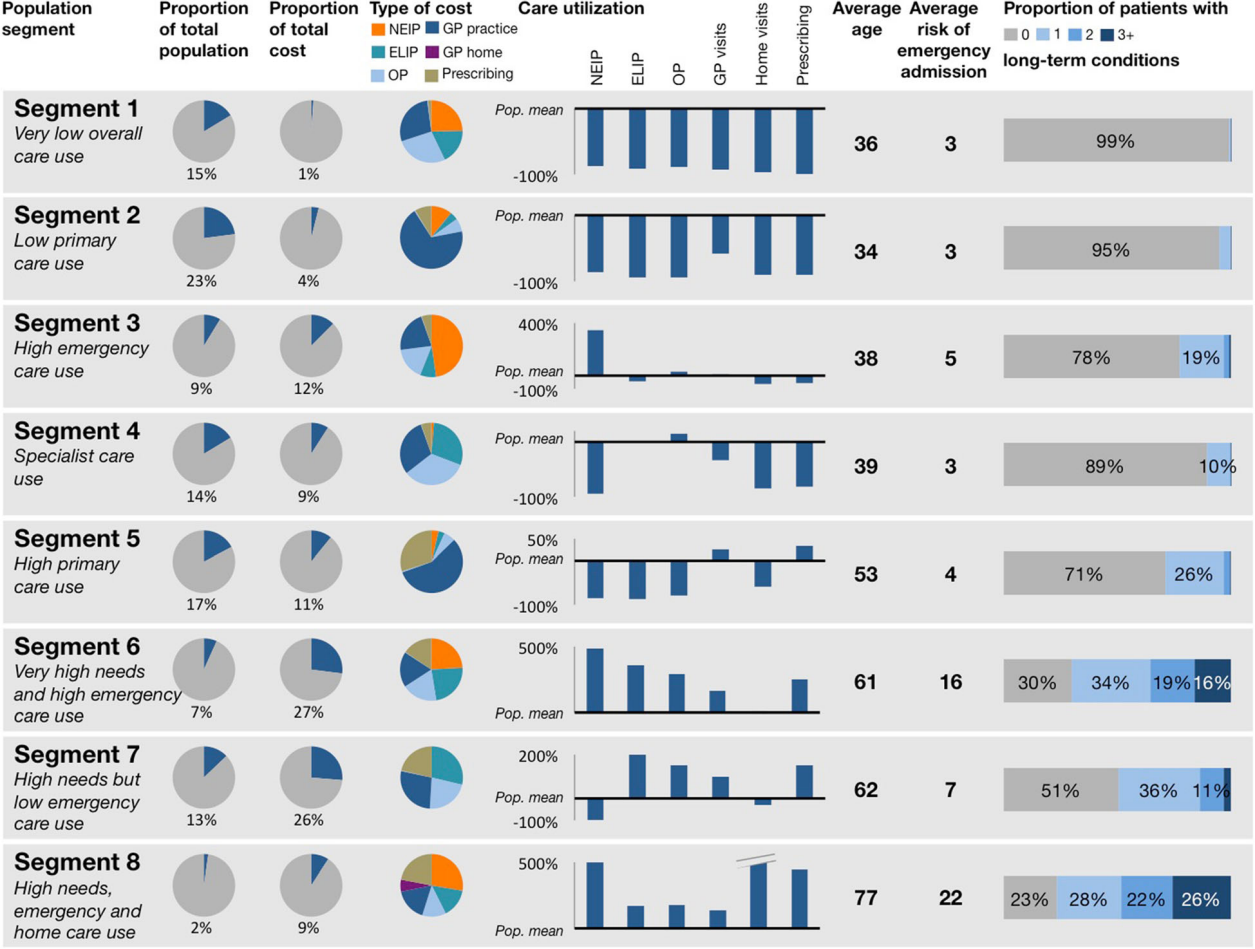
Care user segment profiles. Segment size and cost as a proportion of the total population; segment cost split by care type (NEIP: Non-elective inpatient; ELIP: Elective inpatient; OP: Outpatient; GP practice visits, GP home visits and prescribing); relative care utilization (percentage difference from the overall population mean (Pop. mean) – y-axes vary); average segment age (based on age at the end of the study period); average risk score (risk of an emergency admission in 2012 as a percentage, as predicted based on 2008–2011 data); and the distribution of the number of long-term conditions (LTCs) among patients in the segment

By comparing the cluster mean to the overall population mean, cluster profiles were created that describe distinct care user segments (see Fig. 1). Segments one and two are both low-needs segments. Patients in segment one have very low overall care use. The young people in this segment have few contacts with the health system over time and account for only a fraction of total cost. Segment two is similar to segment one in terms of age and low care use, but patients in this segment require some primary care. This is correlated with a slightly higher long-term condition count.

Segments three, four, and five all have a higher than average use of specific care settings. Segment three is characterized by a high use of emergency care. This drives the overall cost of this segment up to 12% of the population total. People in segment four are relatively low users of primary and emergency care, but use more outpatient and elective care services. Cancer and asthma make up the majority of the disease burden in this segment. The people in segment five use less acute care than average but see their GP often and require more prescriptions. This is the second-largest segment with 17% of the population.

Segments six, seven, and eight are the high-needs segments, with older patients and higher cost than segments one through five. People in segment six have very high utilization of all care settings. Compared to segment seven, which is of a similar age, segment six has a high burden of cardiovascular conditions such as acute myocardial infarction (AMI), congestive heart failure (CHF), and cerebrovascular disease. While this segment is the second smallest at 7% of the population, it accounts for over a quarter of overall cost. Segment seven has a higher than average usage of primary and elective care services, similar to segment six. However, there is a marked difference, as this group has the lowest number of non-elective admissions of all segments.

With an average age of 77, segment eight has the oldest patients. While all morbidities are more common for this group than in the average population, organic brain conditions such as dementia and cerebrovascular disease are particularly prevalent. In addition, this is the principal segment requiring home visits and residential care. While the segment is very small and contains only 2% of the population, it accounts for 9% of the total cost.

### Care user segments compared to traditional population groups

**Fig. 2:**
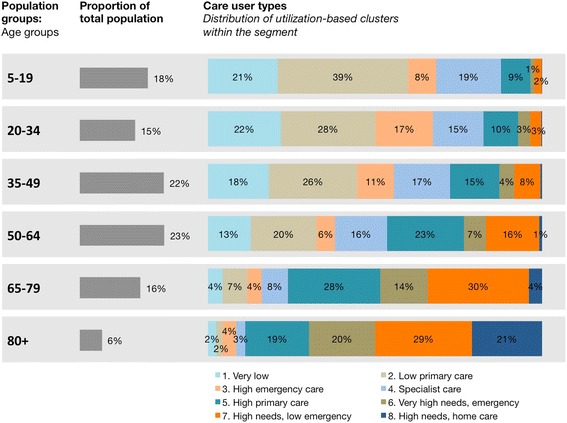
Age groups versus care user segments

Segmenting a population based on age by creating age groups results in roughly equal-sized segments (see Fig. 2). However, each age group is made up of a large variety of care user types, as defined by the utilization-based cluster analysis. Even in the oldest segment of over-80-year-olds, where overall high care needs would be expected, all eight care user segments are represented. In the younger age groups, high-needs segments such as six, seven, and eight are less common. However, this does not mean that care needs are uniform. Instead, the younger age groups consist of a variety of different lower-needs segments.

**Fig. 3:**
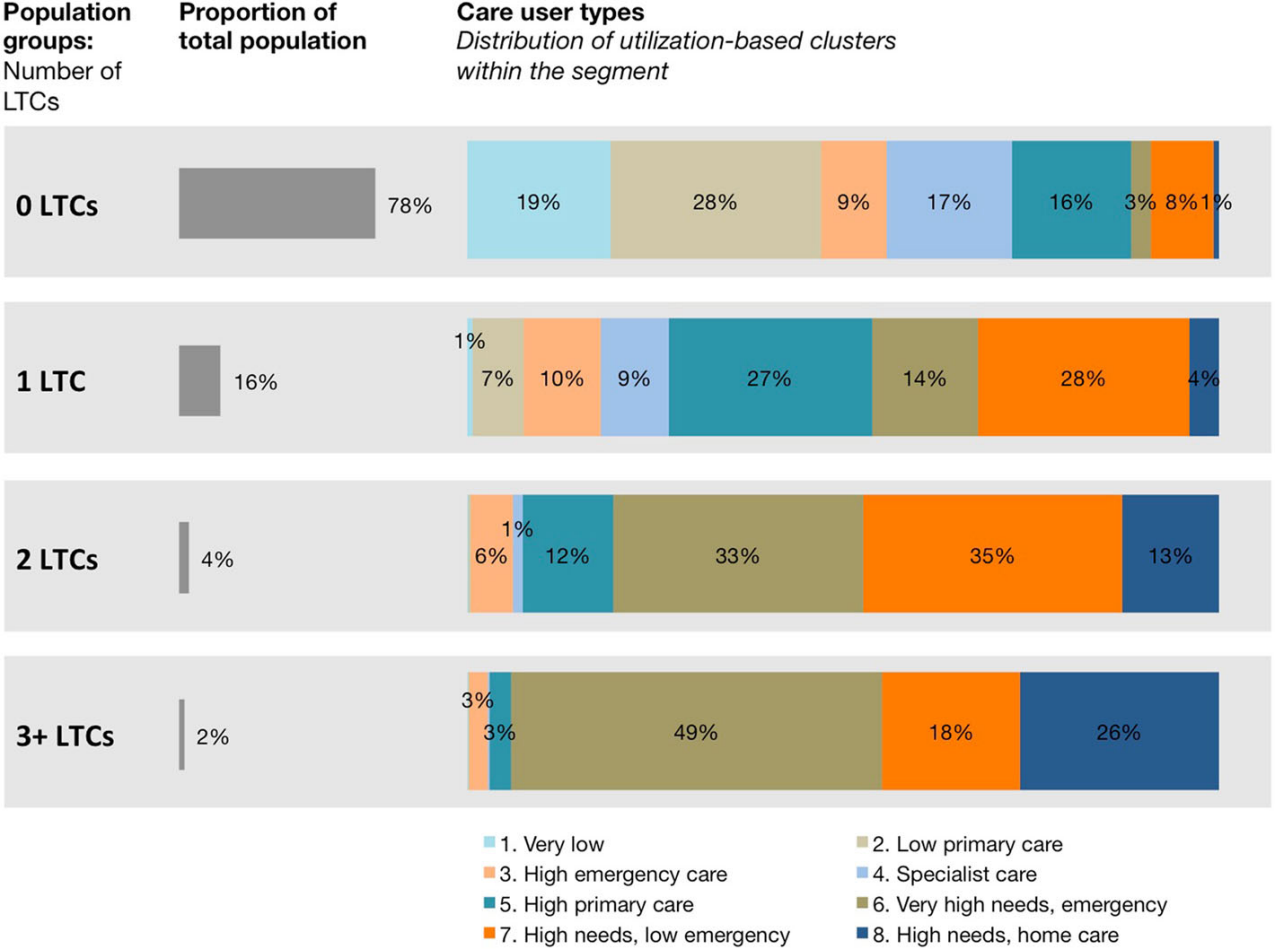
Long-term condition groups versus care user segments

Segmenting a population based on the number of long-term conditions can be considered a proxy for care needs (see Fig. 3). Patients with two long-term conditions are predominantly of care user type six, seven, or eight, all of which are high-need segments. For the group with three or more long-term conditions this effect is even stronger, with over 90% of patients in one of the high-needs segments. However, for the large majority of the population, segmenting on long-term condition count provides little differentiation. In this population, 78% of people do not have a long-term condition and end up in one large segment. The care user segmentation shows, however, that the patients in this large segment do not have homogenous care needs but span all eight care user types.

## Discussion

### Using utilization-based cluster analysis to create distinct patient segments

Eight unique care user types were identified through a cluster analysis of utilization variables. Although the segments were based only on utilization patterns, they each presented with unique morbidity patterns and demographic characteristics. This allows for population health and care priorities to be identified for each segment.

Segments one and two both have a low long-term disease burden, making preventive care a key priority to maintain their health and avoid future cost. However, since people in segment one have little to no contact with the health system, they will need to be targeted via non-health care routes. For segment three the focus should be on prevention of acute episodes, potentially using specialized risk prediction models that can identify patients in this segment. Segment four may benefit from multispecialty community providers as defined in the NHS Five Year Forward View, to integrate some of their outpatient care with the ambulatory setting [19]. Systematic care and support planning can improve outcomes for patients with chronic conditions in segment five and prevent complications [20].

Segments six, seven, and eight all require more intensive case management to coordinate the different care services they use. Case managers can also educate patients on self-management, which is complicated by the high rates of multimorbidity in these segments [21]. For segments six and eight, preventing further complications and emergency admissions is crucial. Segment seven, however, has very few emergency admissions, and interventions for this group should help manage existing conditions and maintain vitality. In addition, segment eight could benefit from comprehensive geriatric assessments [22] and medicine optimization to avoid adverse drug reactions from their large number of prescriptions [23]. However, the reduced mobility of this segment, as reflected by home visits and residential care use, should be considered when designing and implementing these interventions.

### Comparing utilization-based cluster analysis to traditional population groups

Comparing the segments identified through cluster analysis to traditional population groups shows that, at a high level, traditional methods are able to identify higher-needs patients. The groups with three or more long-term conditions and with over-80-year-olds consist mainly of the high-needs segments six, seven, and eight. Similarly, at the other end of the population, lower-needs segments were more common.

Nevertheless, two important advantages of utilization-based segmentation can be observed. Firstly, segmentation based on utilization can identify differences between lower-needs population groups. While long-term condition counts allow policymakers and providers to identify a small group of high-needs patients, they do not provide useful information at the other end of the spectrum. There exists a great diversity of care needs in people without multimorbidity, to which these traditional segmentation methods provide no insight. Although the high-needs patient are very costly, the lower-needs segments make up around 80% of the population and are the prime target for prevention programs. It is therefore crucial that a population health strategy is able to consider the disparate care priorities of this large group.

Second, the care user types identified by utilization-based segmentation provide a perspective on cross-setting care needs. At a high level, traditional methods correspond with low- and high-needs segments, but they fail to differentiate between different types of needs. Segments three and five have similar morbidity counts, but while segment five manages their conditions with primary care services, segment three has a very high chance of needing emergency hospital care. Similarly, segments six and seven are of similar age, but patients in segment six have nearly double the number of long-term conditions and require frequent emergency care. These different types of care users have different population health requirements in terms of disease management, prevention, and education. While utilization is still only a proxy for actual needs, it provides more detailed insight into the care requirements of the different population groups.

### Policy implications

A data-driven population segmentation as demonstrated in this paper can produce novel insights that support evidence-based decisions on population health. Policymakers can use this type of analysis to develop a population health strategy that considers both care and prevention, includes the entire population, and delivers interventions tailored to the segments’ needs.

In addition to providing an evidence base for population health, planning at a segment level rather than for specific diseases or providers can also help the integration of care. When policies or budgets are set by care setting, or for specific conditions, care models that aim to integrate care delivery across these silos will be obstructed. Instead, plans and budgets should be based around segments of patients with similar needs, measured across care settings and regardless of the type of condition, to enable integration.

While this paper focuses on the advantages of utilization-based segmentation, it can be used to complement traditional methods. For example, applying utilization-based cluster analysis to a specific disease population can provide in-depth insights into condition-specific care needs and inform capitated payment schemes or care plans. Alternatively, segmenting the elderly population based on utilization can support the design of more tailored interventions for this group.

Importantly, for any segmentation to be used in practice, linked healthcare datasets need to be available. To make informed decisions on population health, patient-level information is needed for each setting to understand the patient pathway across the care continuum. Unfortunately, access to these types of datasets is still limited [24]. Policymakers should consider the range of levers at their disposal to encourage and facilitate the use of data in health care, such as legislation, investing in the right capabilities, and leading by example. In addition, policymakers could support adoption of these methods by investing in the development and dissemination of technological guidance and software.

### Limitations and further research

An important limitation of this research, in addition to the well-documented general limitations of using administrative data [25, 26], is its limited scope in terms of care settings. The Accident and Emergency (A&E), or emergency department, plays an important part in understanding patient pathways from primary to secondary care, but data for this care setting were not yet available in a linked format to CPRD primary care data. In addition, data on mental health services could significantly improve the understanding of overall care needs. A relatively high prevalence of mental health conditions in segment three suggests the use of acute hospital care for mental health emergencies, but more detailed data are required to explore this further. Furthermore, if social care data could be linked to medical care records, segmentation could provide an evidence base to support the widespread move to integrate the two.

Another limitation of this research is that it is based on a random selection of GP - registered patients from across England. While GP registration is close to universal in England [27], it does leave a small group of patients with distinct care needs out of scope. In addition, the results presented here may not be reflective of local patterns. This applies in particular to more deprived populations, which may see significantly higher rates of multimorbidity [28]. Analysis of local population datasets would likely show different segment sizes and possibly even different segment types, depending on the health status of the community. While we believe the segments described in this paper can be used for high-level strategy decisions, we recommend that local health care commissioners and providers run their own analyses for detailed planning.

## Conclusion

This analysis shows how population segmentation, and in particular a data-driven approach based on utilization variables, can provide a quantitative overview of a population’s care needs to support population health strategies. Segmenting the population based on a cluster analysis of utilization variables creates a multidimensional picture of care needs, cutting across traditional silos such as care settings and disease groups. Long-term condition counts and age groups can be used to identify the small group of high-needs patients in a population but provide little useful information for primary and secondary prevention. Utilization-based cluster analysis, on the other hand, segments the entire population into meaningful groups with unique care priorities, creating an evidence base for whole population health strategies.

## Additional files

Additional file 1: Database construction methods. (DOCX 52 kb)
Additional file 2: Evaluation of the number of clusters. (DOCX 612 kb)

## Abbreviations

A&E: Accident and Emergency
AMI: Acute myocardial infarction
CHF: Congestive heart failure
CPRD: Clinical Practice Research Datalink
ELIP: Elective inpatient admissions
GP: General practitioners
HES: Hospital Episode Statistics
LTCS: Long-term conditions
NEIP: Non-elective inpatient admissions
OP: Outpatient
