# Supplementary material for: A quantitative evidence base for population health: applying utilization-based cluster analysis to segment a patient population
Source: Popul Health Metr. 2016 Nov 25;14:44. doi: 10.1186/s12963-016-0115-z (PMC5124281; doi:10.1186/s12963-016-0115-z)
Supplement: Additional file 1: — Database construction methods. (DOCX 52 kb) [file 12963_2016_115_MOESM1_ESM.docx]

# Additional file 1: Database construction methods

## Population sample selection criteria

To ensure a complete care record, patients were included if they had an active registration with a CPRD-participating primary care practice during the entire study period (2008 up to and including 2012), and if their CPRD records could be linked to HES.

## Utilization variables

The variables were measured over a five-year study period, to account for random variation, but are presented as an annualized mean value in this paper. The highly skewed nature of health care utilization, with a few patients accounting for a large proportion of care, can complicate the clustering process. We therefore excluded extreme high utilizers (in the top 0.1% for any utilization variable), resulting in the exclusion of 0.5% of patients from the sample. In addition we log-transformed the variables to increase the weight of lower numbers, and applied a min-max normalization to put equal weight on each variable.

Utilization variables were defined according to common NHS definitions for elective inpatient admissions (including regular attenders) and non-elective admissions.^1,2^ In the primary care dataset, activity counts were calculated for surgery consultations, telephone contacts, and home visits, as derived from the type of consultation, COT, code in CPRD. These activities were included in the dataset to calculate cost, since their specific unit costs are available from the Unit Cost Health And Social Care study.^3^ For care utilization, only face-to-face interactions are considered, and surgery and clinic visits are combined as they reflect similar types of care. For both prescriptions and outpatient attendances the total number was calculated.

## Long-term conditions

The number of long-term conditions (LTCs) was calculated from both primary and acute care data. A condition was flagged if the patient had a diagnosis in at least one dataset anytime during the five years. The list of conditions was based on the diseases constituting the Carlson Comorbidity Index,^4^ supplemented with any diagnosed mental health conditions or learning disabilities.

The Charlson Index has been proven to work using ICD-based diagnosis information from administrative data, and is one of the most widely used comorbidity indices.^5-8^ Moreover, while it is originally an index predicting mortality,^4^ it has been shown to also correlate with avoidable hospital admissions,^6^ health-related quality of life,^9^ and health care cost.^10^ The Charlson Index combines 16 conditions and assigns them numerical standard weighting to create the overall score.^4^ This study, however, uses the individual conditions specified by the Charlson Index as variables rather than their combined score, to enable the exploration of different patterns at the condition level. There exist different versions of ICD-10 translations of the original ICD-9 codes.^6,8,11-14^ This research uses the translation developed by Aylin at al.^14,15^ because it has been adapted to English coding practices and population characteristics, and because it is used by the NHS Health and Social Care Information Centre in statistical guidance to NHS institutions.^13^ A translation to READ codes has also been created specifically for use in datasets like CPRD, and is based on the General Practice Research Database.^16^

For the purpose of this study, the condition rather than its state (e.g., diabetes, versus diabetes with complications) is used, as patient characteristics should be the same over time. Therefore “diabetes” and “diabetes with complications” are combined, as well as “mild liver disease” and “severe liver disease,” and “cancer” and “metastatic cancer.”

While mental health conditions have not been included in the Charlson index, they have a significant impact on a patient’s care needs, such as higher overall utilization of care,^17^ more unplanned and potentially preventable admissions,^18^ and more readmissions.^19^ This study uses the coding as defined by White et al.^20^ as it provides a wide definition for psychosis and bipolar disorders that includes Chang et al.’s^21^ criteria as well as those of NHS England when excluding drug-induced and depression-related psychosis (these may be temporary states rather than enduring mental illnesses and should therefore be excluded).^22^

Learning disabilities are another group of conditions that significantly impact a person’s care needs, but that are not included in general morbidity indices. Like mental health, they are included in the NHS Quality and Outcomes framework,^23^ but no specific conditions are listed for this metric. While in England the term learning disabilities is common, the World Health Organization uses “mental retardation.”^24^ This group of conditions is covered by ICD-10 codes F70-F79.^25^

## Cost

The costs for acute care are derived from NHS Reference Costs 2011/12.^26^ While the actual cost of treating patients may vary by provider,^2,27^ the aim is to give a relative indication of cost to identify and compare high-cost individuals. The Reference Costs provide a national average cost based on the HRG code for the spell, taking into account the type of admission (day case, elective, non-elective or regular attender). Non-elective cases are assigned different cost for short stays (less than two days) and long stays (two days or longer).^28^ For admissions without an HRG code in the database, admissions from 2008 before the introduction of HRG version 4.0, and regular attender visits, a weighted average cost was calculated for each activity type from the NHS Reference Costs. Costs for outpatient attendances were based on a weighted average from the NHS Reference Cost, calculated per treatment specialty.

Similar as for acute care, prescription costs are based on Net Ingredient Cost (NIC) rather than actual cost, disregarding any local fees or discounts. The CPRD database holds information on the British National Formulary (BNF) cost for each prescription; however, these differ slightly from those used by Health and Social Care Information Centre (HSCIC) at the 5th, 6^th^, and 7th digit due to the recording systems allowing additional splits at this level (CPRD Knowledge Centre, personal communication, 21 April 2015).

To estimate average NIC per prescription for each prescription, Prescription Cost Analysis (PCA) data for 2012 from the HSCIC are used.^29^ This dataset contains cost and volume information on drugs prescribed in England for each 15-digit BNF code. The data were aggregated at both the 6-digit and 4-digit levels, calculating weighted average cost for each. If no 6-digit match was available in the CPRD dataset due to coding differences, a 4-digit match would provide the unit cost instead.

For primary care activities unit costs were estimated from the Unit Cost Health and Social Care.^3^ The Unit Cost report calculates cost for GP services by comparing salary, overheads, and other costs for the practice to the activities performed, taking into account the amount of time each activity takes. Separate unit costs for clinic, surgery, and telephone consultations, as well as home visits, are available and used in our analysis.

## Risk score

Risk prediction models can be created for any type of outcome, but many systems use emergency admissions or readmission.^30-32^ We calculated our own risk prediction score, based on a range of predictor variables from PARR, the Combined Predictive Model, and other risk prediction algorithms.^30,33-35^ We trained the risk model to predict emergency admissions in 2012 using a stepwise logistic regression (backward, likelihood ratio), with the number of emergency admissions in 2011 as one of the predictor variables, as well a range of other variables including specific diagnoses, utilization of outpatient and prescribing services, deprivation, age, and gender. Diagnoses and utilization variables are measured over 2008–2011 to reflect the information that is used for the segments (which is based on 2008–2012) as closely as possible.

To validate the model, we used a split sample validation method, with half of the sample functioning as the training set and the other half as the test set. The area under the Receiver Operator Curve (ROC) for the test set was 0.75, which is in line with other risk prediction studies, which range from 0.55 to 0.83.^30,34^

1. Department of Health. *NHS Reference cost 2012-13.* London: Department of Health; 2013.

2. Department of Health. *Step-by-Step guide: Calculating the 2013-14 National Tariff.* London: Department of Health; 2013.

3. Curtis L. *Unit Cost of Health and Social Care 2013.* Kent: Personal Social Services Research Unit; 2013.

4. Charlson ME, Pompei P, Ales KA, MacKenzie CR. A new method of classifying prognostic comorbidity in longitudinal studies: Development and validation. *Journal of Chronic Diseases.* 1987;40(5):373-383.

5. de Groot V, Beckerman H, Lankhorst GJ, Bouter LM. How to measure comorbidity: a critical review of available methods. *Journal of Clinical Epidemiology.* 2003;56(3):221-229.

6. Halfon P, Eggli Y, van Melle G, Chevalier J, Wasserfallen J-B, Burnand B. Measuring potentially avoidable hospital readmissions. *Journal of Clinical Epidemiology.* 2002;55(6):573-587.

7. Deyo RA, Cherkin DC, Ciol MA. Adapting a clinical comorbidity index for use with ICD-9-CM administrative databases. *Journal of Clinical Epidemiology.* 1992;45(6):613-619.

8. Sundararajan V, Henderson T, Perry C, Muggivan A, Quan H, Ghali WA. New ICD-10 version of the Charlson comorbidity index predicted in-hospital mortality. *Journal of Clinical Epidemiology.* 2004;57(12):1288-1294.

9. Fortin M, Hudon C, Dubois MF, Almirall J, Lapointe L, Soubhi H. Comparative assessment of three different indices of multimorbidity for studies on health-related quality of life. *Health and quality of life outcomes.* 2005;3:74.

10. Perkins AJ, Kroenke K, Unutzer J, et al. Common comorbidity scales were similar in their ability to predict health care costs and mortality. *J. Clin. Epidemiol.* 2004;57(10):1040-1048.

11. Quan H, Sundararajan V, Halfon P, et al. Coding algorithms for defining comorbidities in ICD-9-CM and ICD-10 administrative data. *Medical care.* 2005;43(11):1130-1139.

12. Aylin P, Bottle A, Jen MH, Middleton S. *HSMR mortality indicators.* Leeds: NHS England; 2009.

13. NHS Information Centre for Health and Social Care. *Indicator specification: Summary Hospital-level Mortality Indicator.* Leeds: NHS Information Centre for Health and Social Care; 2014.

14. Aylin P, Bottle A, Jen MH, Middleton S. *HSMR mortality indicators.* Dr Foster Unit, Imperial College; 2010.

15. Bottle A, Aylin P. Comorbidity scores for administrative data benefited from adaptation to local coding and diagnostic practices. *Journal of Clinical Epidemiology.* 2011;64(12):1426-1433.

16. Khan N, Perera R, Harper S, Rose P. Adaptation and validation of the Charlson Index for Read/OXMIS coded databases. *BMC Family Practice.* 2010;11(1):1.

17. Doherty AM, Gaughran F. The interface of physical and mental health. *Social psychiatry and psychiatric epidemiology.* 2014;49(5):673-682.

18. Payne RA, Abel GA, Guthrie B, Mercer SW. The effect of physical multimorbidity, mental health conditions and socioeconomic deprivation on unplanned admissions to hospital: a retrospective cohort study. *CMAJ : Canadian Medical Association journal = journal de l'Association medicale canadienne.* 2013;185(5):E221-228.

19. Bottle A, Aylin P, Bell D. Effect of the readmission primary diagnosis and time interval in heart failure patients: analysis of English administrative data. *European journal of heart failure.* 2014;16(8):846-853.

20. White J, Gutacker N, Jacobs R, Mason A. Hospital admissions for severe mental illness in England: Changes in equity of utilisation at the small area level between 2006 and 2010. *Social science & medicine.* 2014;120C:243-251.

21. Chang C-K, Hayes RD, Perera G, et al. Life Expectancy at Birth for People with Serious Mental Illness and Other Major Disorders from a Secondary Mental Health Care Case Register in London. *PloS one.* 2011;6(5):e19590.

22. NHS England. *CQUIN 2014/25 - additional guidance on the national mental health indicator.* Leeds: NHS England; 2014.

23. NHS Information Centre for Health and Social Care. Quality and Outcomes Framework (QOF) for April 2012 - March 2013, England - Disease prevalence. 2013.

24. Royal College of Psychiatrists’ Faculty of Psychiatry of Intellectual Disability. *People with learning disability and mental health, behavioural or forensic problems: the role of in-patient services.* London: Royal College of Psychiartists; 2013.

25. ICD10 Online application 2014. <http://apps.who.int/classifications/icd10/browse/2010/en>. Accessed 20 October 2014.

26. Department of Health. National Schedule of Reference Costs 2011-12 for NHS trusts and NHS foundation trusts. London: Department of Heath; 2012.

27. Department of Health. *A simple guide to Payment by Results.* London: Department of Health; 2012.

28. Department of Health. *Reference cost 2011-12.* London: Department of Health; 2012.

29. NHS Health and Social Care Information Centre. Prescription Cost Analysis, England - 2012. Leeds: NHS Health and Social Care Information Centre; 2013.

30. Kansagara D, Englander H, Salanitro A, et al. Risk prediction models for hospital readmission: A systematic review. *JAMA.* 2011;306(15):1688-1698.

31. Love T, Swansson J, Whelen C. *Development of an algorithm to stratify patients by risk of acute hospitalisation.* Auckland: Sapere; 2014.

32. NHS England. *Using case finding and risk stratification: A key service component for personalised care and support planning.* Leeds: NHS England; 2015.

33. Billings J, Dixon J, Mijanovich T, Wennberg D. Case finding for patients at risk of readmission to hospital: development of algorithm to identify high risk patients. *Br. Med. J.* 2006;333:327.

34. Billings J, Blunt I, Steventon A, Georghiou T, Lewis G, Bardsley M. Development of a predictive model to identify inpatients at risk of re-admission within 30 days of discharge (PARR-30). *BMJ Open.* 2012;2(4).

35. Wennberg D, Siegel M, Darin B, et al. *Combined Predictive Model - Final report & technical documentation.* London: Health Dialog, King's Fund and New York University; 2006.
