# Supplementary material for: A quantitative evidence base for population health: applying utilization-based cluster analysis to segment a patient population
Source: Popul Health Metr. 2016 Nov 25;14:44. doi: 10.1186/s12963-016-0115-z (PMC5124281; doi:10.1186/s12963-016-0115-z)
Supplement: Additional file 2: — Evaluation of the number of clusters. (DOCX 612 kb) [file 12963_2016_115_MOESM2_ESM.docx]

Additional file 2

Evaluation of the number of clusters

A k-means method was used to segment the population because this method can handle large sample sizes.^1^ However, this method requires the user to determine the number of clusters (k) before the analysis. To identify how many clusters exist in the data, 10 samples of 3,000 patients were analyzed using a hierarchical clustering approach (Ward’s), which can be used to identify k.

Across the samples, 7- to 10-cluster solutions were found. Ward’s method is sensitive to outliers, and this may have caused the varying results across samples.^2^ Despite the log-normalization of the clustering variables, there still exist a large number of outliers (see Figure S1). Especially in the smaller samples used for the clustering, these outliers could have changed the resulting clusters.

Figure S1: Box plots of the standardized, log-normalized clustering variables


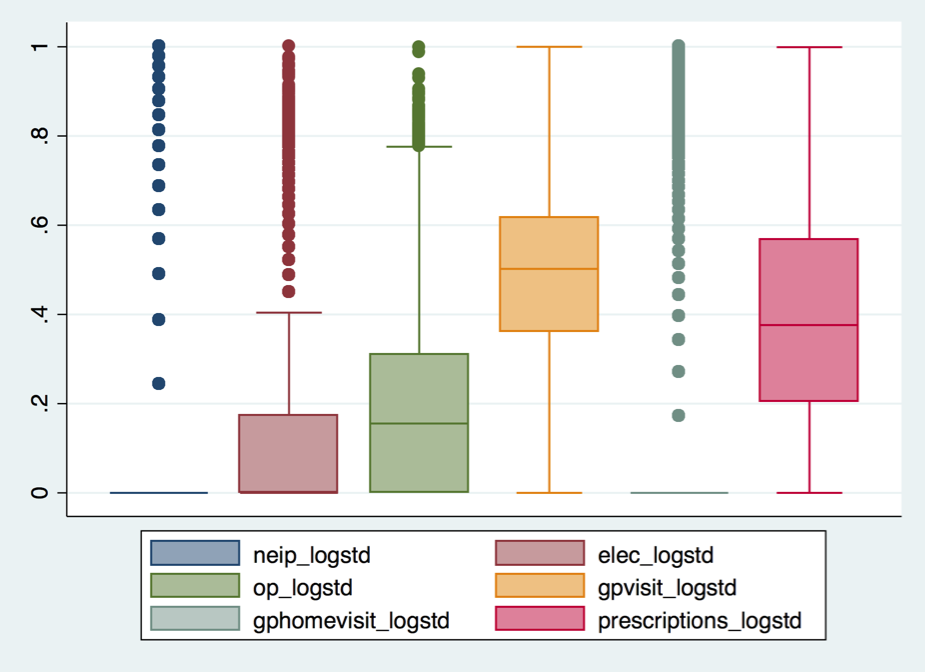


However, in cluster analysis there is no absolute “right” answer.^3^ The optimal number of clusters will depend on the practical use of the segments, such as their interpretability, actionability, and ease of use.^4^ Therefore, the k-means method was applied to the whole population for 7, 8, 9, and 10 clusters. The results of these cluster analyses were then compared for their usefulness (see Figure S2).

Figure S2: Practical comparison of the various cluster solutions

The 8-cluster solution improved on the 7-cluster model by splitting a high-needs cluster into two distinctive groups: one with, and one without emergency hospitalizations. Practically, these two groups are very different as one appears to have acute complications or exacerbations, while the other group manages their complex care needs with elective and primary care. The 9-cluster solution, however, did not create any clinically relevant additional segments, instead identifying a group of patients with lower to average care needs. These patients do no form a distinct subgroup of one of the 8-cluster groups, thus not revealing any unexposed patterns within a cluster. Based on these analyses, the 8-cluster solution was selected.

The cluster validity was confirmed post-hoc by running a split-sample analysis, where the dataset was split into two equal-sized groups and both were analyzed with a k-means analysis for 8 clusters. The results show that the same segments were found in both subsets as in the overall population, confirming that the clusters were not a random chance occurrence (see Figure S3).

Figure S3: Split-sample analysis of the 8-cluster solution

**References**

1. Han J, Kamber M. *Data Mining: Concepts and Techniques*. 1st ed. San Diego, CA: Academic Press, 2001.

2. Everitt BS, Landau S, Leese M, et al. *Cluster analysis*. 5th ed. Chichester: John Wiley & Sons, 2011.

3. Han J, Kamber M, Pei J. *Data Mining: Concepts and Techniques*. 3rd ed. Waltham, MA: Morgan Kaufmann, 2011.

4. Tsiptsis K, Chorianopoulos A. *Data Mining Techniques in CRM: Inside Customer Segmentation*. Chichester: John Wiley & Sons, 2009.
